# Energetic Coordination Compounds: Investigation of Aliphatic Ligands and Development of Prototype Detonators

**DOI:** 10.3390/ijms25168645

**Published:** 2024-08-08

**Authors:** Klaudia Pawlus, Agnieszka Stolarczyk, Tomasz Jarosz, Mateusz Polis, Konrad Szydlo, Łukasz Hawełek, Sylwia Waśkiewicz, Mieczysław Łapkowski

**Affiliations:** 1 Department of Physical Chemistry and Technology of Polymers, Silesian University of Technology, 44-100 Gliwice, Polandsylwia.waskiewicz@polsl.pl (S.W.);; 2 Explosive Techniques Research Group, Łukasiewicz Research Network—Institute of Industrial Organic Chemistry, 42-693 Krupski Młyn, Poland; 3 Lukasiewicz Research Network—Institute of Non-Ferrous Metals, 44-100 Gliwice, Poland

**Keywords:** energetic coordination compounds, transition metal coordination compounds, application of energetic coordination compounds in detonators

## Abstract

In this work, energetic coordination compounds (ECCs) of transition metals (Fe, Ni, Cu, Zn) containing aliphatic amines as ligands were synthesized: ethylenediamine; 1,3-diaminopropane; tris(2-aminoethyl)amine; tris(3-aminopropyl)amine. The compounds were investigated in terms of ignition/explosion temperature, friction and impact sensitivity. For selected compounds, structural characterisation was presented (IR-ATR spectroscopy, Raman spectroscopy) and their morphology was determined (SEM, powder XRD). They were also investigated by differential scanning calorimetry (DSC). In order to assess the potential application of selected ECCs in detonators, underwater explosion tests were carried out, determining energetic performance. The results achieved for detonators containing ECCs were compared with those for reference detonators (containing pentaerythritol tetranitrate, PETN), indicating their potential use as a “green” alternative to nitric acid esters.

## 1. Introduction

Detonators are devices that are the initial source of detonation in virtually all blasting operations. These devices contain a small amount (approx. 0.8–1.2 g) of a primary energetic material (EM) (most commonly lead azide or lead styphnate), as well as a secondary EM and one or more pyrotechnic compositions. The annual scale of production of detonators was reported in 2014 as 3.7 billion for China alone [1] and the use of detonators increases by several percent annually. This translates into thousands of metric tonnes of lead compounds being introduced into the environment each year. Moreover, the manufacture of lead azide requires the use of sodium azide as a raw material, carrying with it another toxicological and environmental hazard.

The use of lead salts in detonators is being tolerated for the sole reason of a lack of viable material alternatives, with prospective replacements for lead salts showing either insufficient performance or being extremely cost-intensive. This state of affairs has given rise to extensive research on new primary EMs [2], based on aliphatic compounds [3,4,5,6,7] and heterocyclic compounds [8,9,10,11,12]. Among these groups, coordination compounds offer the highest amount of degrees of freedom in design, as well as allow the use of relatively standard raw materials of the chemical industry as sources of the central atoms, ligands and counterions.

Early work on the development of energetic coordination compounds (ECCs) maintained the approach of using already available raw materials [13], even if the choice of ligands (i.e., hydrazine, ethylenediamine) prioritized energetic performance over lack of hazardous properties.

**Figure 1:**
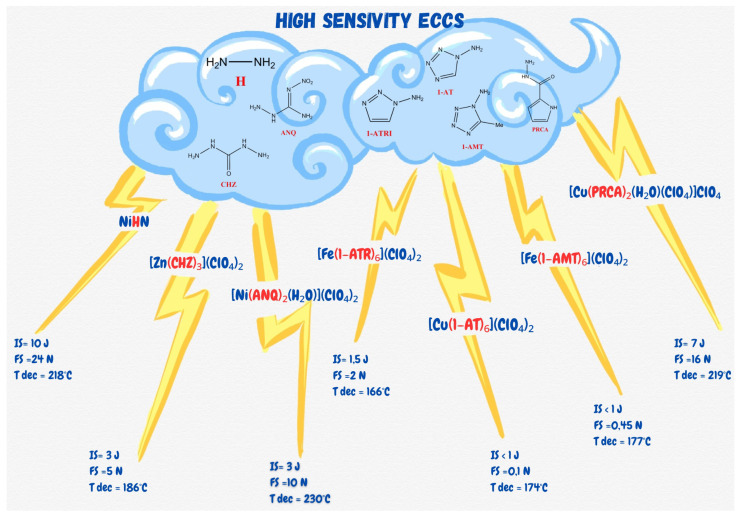
Examples of energetic coordination compounds.

Further work on ECCs has brought about a staggering array [14,15] of prospective materials. Despite the advantageous sensitivity parameters and energetic properties, most of the reported materials require complex chemical synthetic routes to produce the necessary ligand molecules. Not only is such an approach incompatible with the tenets of “green” chemistry, but many of the reported compounds can be seen as equally problematic as lead azide, i.e., ECCs, in which cadmium is the central atom [16]. The former drawback precludes adoption of the “green” material on an industrial scale due to associated costs and the latter makes such adoption undesirable. To provide the Reader with an overview of the current state of the art, a brief summary of the most relevant reported ECCs is presented in Figure 1.

The goal of this work was to develop ECCs that are free of the two above-mentioned drawbacks, being as “green” as possible, while maintaining a unit cost that will not invalidate any application potential. To this effect, we have investigated a series of ECCs produced using relatively simple aliphatic ligands, i.e., difunctional and trifunctional amines. These compounds have been subjected to a range of investigations, revealing their chemical identities, their energetic parameters and providing pre-application data in the form of mock detonators utilizing them in place of the currently used secondary (i.e., pentaerythritol tetranitrate (PETN)) EMs.

## 2. Results and Discussion

### 2.1. Spectroscopic Investigations

The following figures show IR-ATR spectra for compounds Cu-L2-C and Cu-L3-N, comparing the spectrum of the purified and unpurified compound.

**Figure 2:**
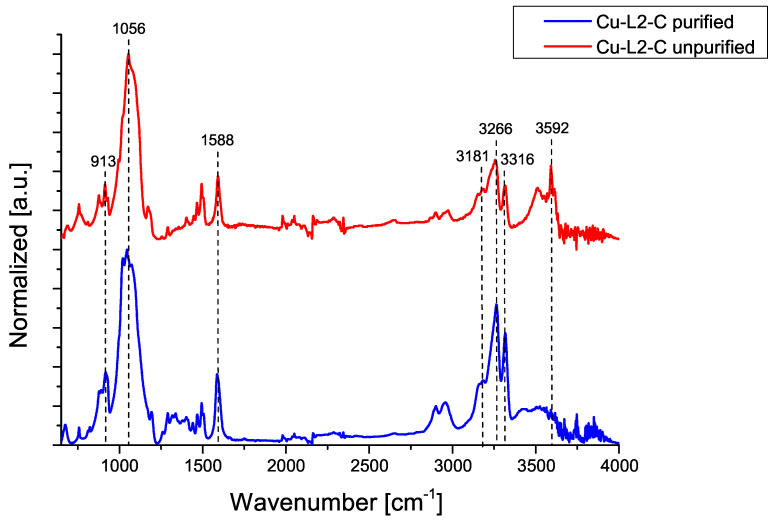
IR-ATR spectra of Cu-L2-C compound before and after purification.

Figure 2 shows a comparison of the IR-ATR spectra of the purified and unpurified Cu-L2-C compound. In both cases, spectra contains signals corresponding to deformation vibrations for N–H in the range of $1500–1650\;{cm}^{-1}($$v=1588\;{cm}^{-1}),$ signal for N–H stretching vibrations in the range of 3200–3600 cm^−1^ (δ = 3266 cm^−1^ and 3316 cm^−1^) are present. C–H bonds are also found in the absorption band for stretching vibration in the range of $2850–3000\;{cm}^{-1}.$ A single absorption band at 1056 cm^−1^ is visible for both the unpurified and purified compound, indicating the presence of a stretching vibration characteristic of the ClO_4_ anion. Also visible is an absorption band for stretching vibration in the range of 3300–3600 cm^−1^ and an absorption band for deformation vibration in the range of 1200–1450 cm^−1^ indicating that O H bonds appear. At 913 cm^−1^ and at 3592 cm^−1^, there are absorption bands indicating the presence of ammonia [17,18].

**Figure 3:**
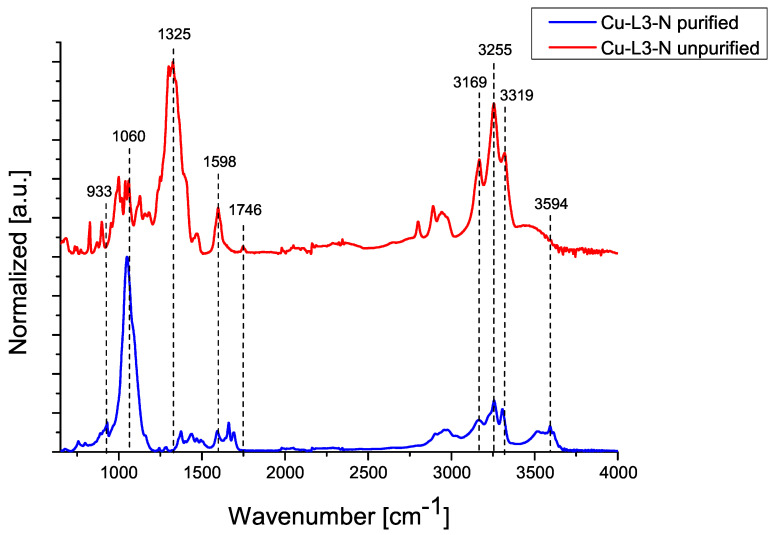
IR-ATR spectra of Cu-L3-N compound before and after purification.

Figure 3 compares the IR-ATR spectra of the purified and unpurified Cu-L3-N compound. In both cases, characteristic absorption bands or N–H deformation vibrations in visible in the range of $1500–1650\;{cm}^{-1}($$v=1598\;{cm}^{-1})$ and absorption bands for N–H stretching vibrations in the range of 3200–3600 cm^−1^ (δ = 3255 cm^−1^ and 3319 cm^−1^) are present. There are also C–H bonds in the absorption band for stretching vibrations in the range of 2850–3000 cm^−1^. In the case of the spectrum of the unpurified sample, it can be seen that at 1746 cm^−1^ there is a characteristic stretching vibration, which indicates the attachment of a water molecule to the ECC molecule. In addition, a single absorption band is visible at 1325 cm^−1^, indicating the presence of a stretching vibration characteristic of the nitrate anion. This indicates the presence of unreacted substrate. This relationship is not present for the purified sample. The absorption band present for stretching vibration in the range of 3300–3500 cm^−1^ and the absorption band for deformation vibration in the range of 1200–1450 cm^−1^ indicates the presence of O–H bonds. Furthermore, peaks at 933 cm^−1^ and at 3594 cm^−1^ indicate the absorption of ammonia [17,18,19,20].

**Figure 4:**
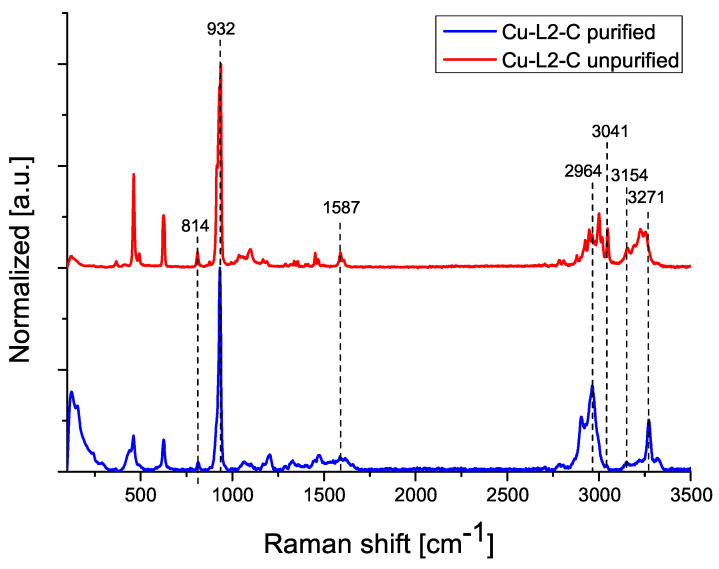
Raman spectra of Cu-L2-C compound before and after purification.

**Figure 5:**
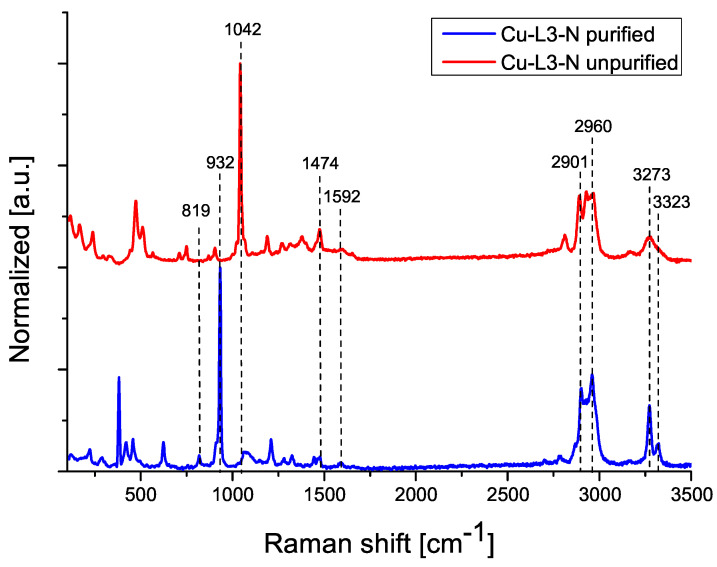
Raman spectra of Cu-L3-N compound before and after purification.

The following figures show Raman spectra for compounds Cu-L2-C (Figure 4) and Cu-L3-N (Figure 5), comparing the spectrum of the purified and unpurified compound.

The Raman spectrum of the Cu-L2-C sample (Figure 4), for both purified and unpurified compounds, contains signals corresponding to the amine group (1587, 2964, 3041 cm^−1^). The signal at 932 cm^−1^ indicates the presence of a ClO_4_ anion. Signals from absorbed ammonia are also present (814, 3271 cm^−1^). The Raman spectrum of Cu-L3-N (Figure 5) contains signals originating from the amine group (1592, 2901, 2960 cm^−1^). For the unpurified sample, the peak at 1042 cm^−1^ indicates the presence of unreacted perchlorate anions. For the purified sample, the signal of perchlorate anions occurs at 932 cm^−1^, indicating the removal of unreacted substrate. Two signals from NH_3_ groups are also visible (819, 3273 cm^−1^) [17,21].

### 2.2. X-ray Diffractometry

**Figure 6:**
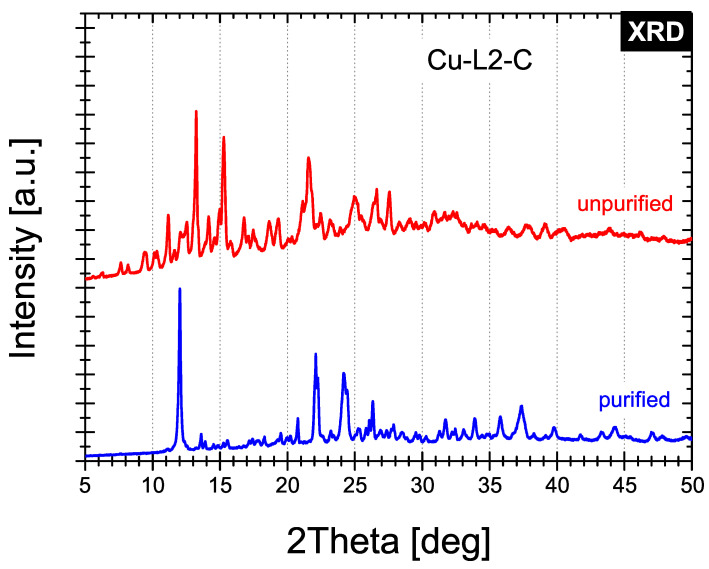
X-ray diffraction patterns of Cu-L2-C before and after purification.

**Figure 7:**
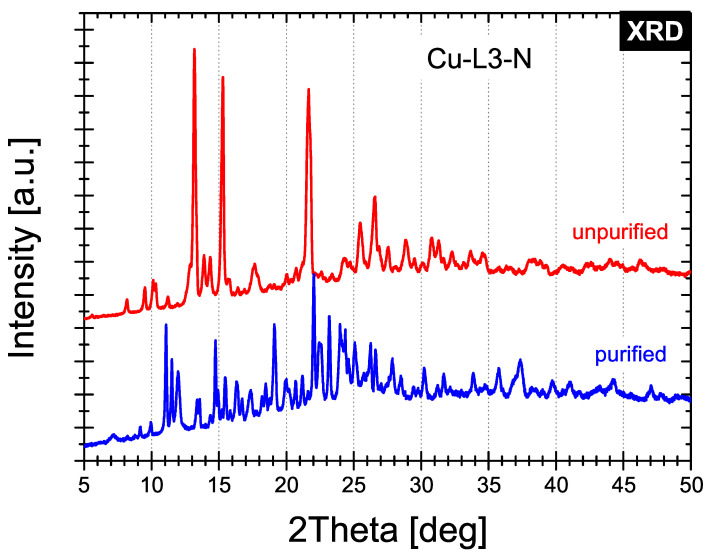
X-ray diffraction patterns of Cu-L3-N before and after purification.

The XRD patterns of the selected synthesised Cu-L2-C and Cu-L3-N compounds are shown in the 2θ range 5°–50° in Figure 6 and Figure 7, respectively.

Analysis of the diffractograms clearly shows that diffraction patterns of the unpurified and purified samples of each compound differ significantly. However, the synthesised samples possess a crystal structure that cannot be identified fully. The presence of only a few Cu-based phases has been identified. For unpurified Cu-L2-C sample four phases have been identified in minority: Cu(ClO_4_)_2_ · 2H_2_O (PDF Card No. 00-032-0329), Cu(ClO_4_)_2_· 4H_2_O (PDF Card No. 01-078-2487), Cu(ClO_4_)_2_· 6H_2_O (PDF Card No. 01-079-0728) and Cu(OH)_2_· H_2_O (PDF Card No. 00-042-0746), while for purified sample two phases may coexist as minor phases: Cu(ClO_4_)_2_· 6H_2_O (PDF Card No. 01-073-1762) and Cu(ClO_4_)_2_ (PDF Card No. 01-083-0976). For the Cu-L3-N compound, only one phase has been identified in the purified counterpart: Cu(NO_3_)_2_· 6H_2_O (PDF Card No. 00-024-0370).

### 2.3. Elemental Analysis

The structure of the purified Cu-L2-C was confirmed by elemental analysis, allowing the content of C, H and N atoms to be determined in the sample (Table 1).

The analysis indicated that the investigated compound contains a minor amount of adsorbed water as well as ammonia.

### 2.4. Morphology Investigation—Scanning Electron Microscopy

**Figure 8:**
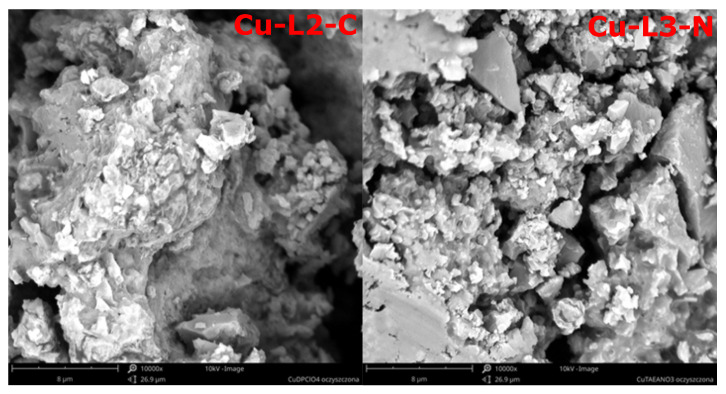
SEM images of the purified Cu-L2-C and Cu-L2-N.

SEM investigations of compounds Cu-L2-C and Cu-L3-N (Figure 8) show that these compounds exhibit an amorphous structure.

### 2.5. Friction and Impact Sensitivity

**Figure 9:**
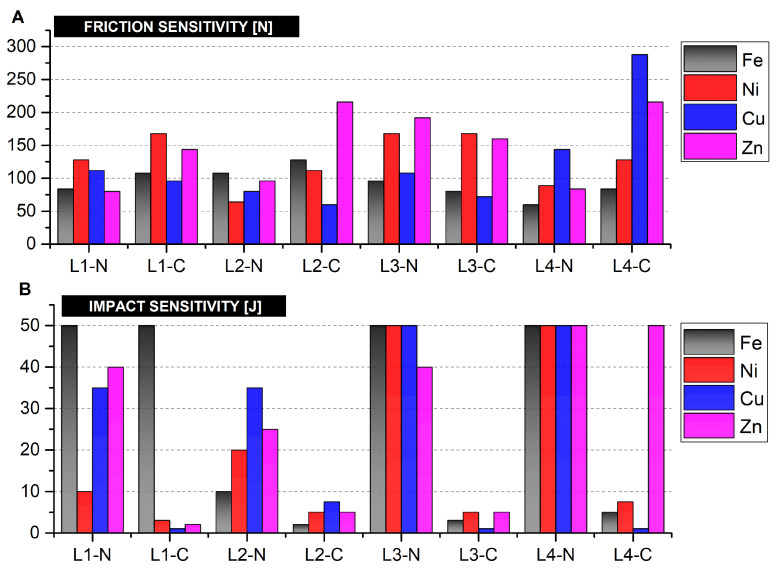
Sensitivity to (**A**) friction; (**B**) impact for the prepared energetic coordination compounds. LX (X = 1–4) indicates the utilised ligand; N or C indicate whether a nitrate (N) or perchlorate (C) anion was present.

The friction and impact sensitivity investigations of the synthesised coordination compounds were conducted to observe whether a dependence of mechanical sensitivity of the ECCs on the employed ligand, metal and oxidising anion (nitrate and perchlorate analogues) exists. Aliphatic amines with either a straight chain (EDA, DP) and containing two amine groups in their structure, or with a branched chain (TAEA, TAPA) and containing three amine groups, respectively, were used as ligands. Considering the friction sensitivity of ECCs (Figure 9), the perchlorate-containing compounds exhibit lower friction sensitivity compared to nitrate-bearing compounds. The exception is compounds containing Cu in the structure, exhibiting higher sensitivity, apart from Cu-L3-C. The detailed sensitivity values of the obtained compounds are given in the appendix (Table A7).

The results of impact sensitivity for obtained compounds are highly varied, ranging from extremely sensitive (for most perchlorate analogues), to insensitive for impact (nitrate analogues, i.e., ECCs containing the L4 ligand with exceptions of Ni-L1-N and Fe-L2-N). Nitrate analogues display negligible impact sensitivity, or are not sensitive at all. The exceptions are Ni-L1-N and Fe-L2-N compounds. In the case of perchlorate analogues, the majority exhibit a sensitivity to impact of less than 10 J and samples with copper reach 1 J or lower, with the exception of Cu-L2-C. In contrast, Fe-L1-C and Zn-L3-C compounds are not sensitive. Despite the lack of a clear dependence of impact sensitivity on the choice of ECC constituents, lesser correlations have been observed, such as a very similar dependence profile of impact sensitivity on the central atom for ECCs containing L2. The dependence of impact sensitivity for L3-C and L4-C ECCs on the choice of central atom was also very similar to each other. This indicates that while the changes in the constituents of the ECCs have an effect on their sensitivity to mechanical stimuli, as expected based on literature (e.g., the nitrogen content of the ECCs), this effect is not straightforward, due to the likely strong influence of the physical form (e.g., grain size and shape, degree of crystallinity) of the produced ECC samples. Regarding the friction sensitivity for the produced compounds, the perchlorate analogues were considered less sensitive, but in turn were more sensitive to impact.

### 2.6. Determination of the Ignition/Explosion Temperature (I/ET)

The results of the ignition/explosion temperature tests are summarised in Table 2. Considering the (I/ET) temperature results obtained, a similar relationship, as in the case of friction and impact sensitivity, is not observable. However, it can be noted that with increasing chain length and degree of branching, the temperature resistance increases. In addition, Zn-containing compounds appeared to be the most thermally resistant, reaching decomposition temperatures of about 300 °C, with a maximum of 362 °C—Zn-L4-C, except for Zn-L1-C (183 °C) and Zn-L3-C (158 °C). On the other hand, the lowest decomposition temperatures were obtained for Fe-containing compounds—158 °C for Fe-L3-C.

The use of perchlorate instead of nitrate as the counter anion can both increase (e.g., for Cu-L1-N vs. Cu-L1-C) and decrease (e.g., for Cu-L2-N vs. Cu-L2-C) the observed IET of the ECCs and may have a minor (9 K for Fe-L2-N vs. Fe-L2-C) or major (122 K for Zn-L3-N vs. Zn-Le-C) impact on this parameter. Similarly, altering the choice of utilised ligand also does not appear to have a uniform effect on the IET values of the investigated ECCs, with the transition to a longer alkyl chain leading to an increase in the IETs of the ECCs with linear ligands (L1 vs. L2), while having a mixed effect on ECCs with branched ligands (L3 vs. L4). The choice of central atom, however, appears to have at least some discernible influence on the IETs of the investigated ECCs. In this case, iron-bearing ECCs consistently showed lower IETs than ECCs bearing other types of central atoms. This may be tentatively attributed to the redox-catalytic properties of iron cations. Similarly, copper- and nickel-bearing ECCs typically were among the more stable thermally (high IET values). Interestingly, Zn-bearing ECCs showed extremely varied IET values (from 158 °C for Zn-L3-C to 364 °C for Zn-L2-C), indicating that the mechanism underlying the thermal decomposition of this group of compounds is not as straightforward as it would be expected based on literature.

Based on the parameters of sensitivity to mechanical and ignition/explosion temperature, two compounds Cu-L2-C and Cu-L3-N were selected for further investigation. In addition, an important aspect in the context of working with the obtained compounds and consequently preparing detonators based on them was their easy processability. The compounds were small-crystalline powders, so they were easy to press into detonators and their low sensitivity parameters did not pose a risk of working with the materials.

### 2.7. Differential Scanning Calorimetry

**Figure 10:**
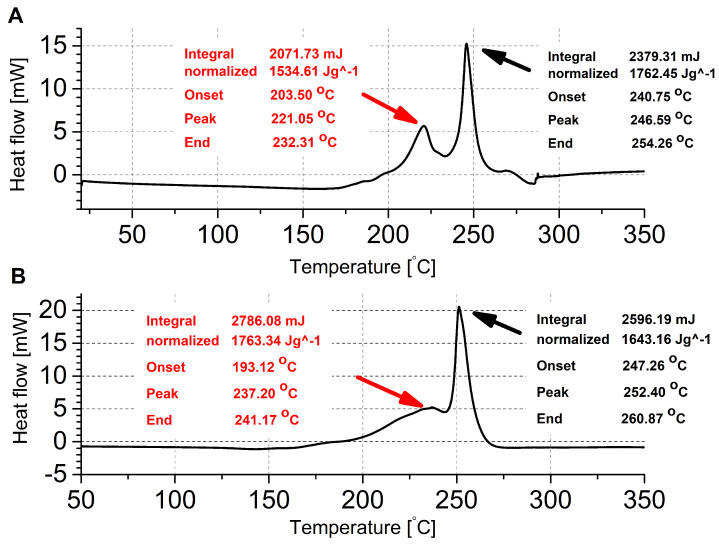
The DSC thermogram recorded for Cu-L2-C (sample mass of 1.30 mg) (**A**) and for Cu-L3-N (sample mass of 1.58 mg) (**B**).

The thermograms (Figure 10) show thermally induced decomposition processes for Cu-L2-C and Cu-L3-N occurring in two stages. During DSC analysis, samples were heated from 20 °C to 350 °C at a heating rate of 5 K/min. In the case of the Cu-L2-C compound, the thermogram show two signals at 221 °C and 247 °C, indicating a two-stage exothermic decomposition process. Similarly, for the Cu-L3-N, the decomposition process also occurs in two stages. The first peak appear at 237 °C and the second at 252.4 °C.

In the thermogram, the presence of the first peak, which overlaps the second peak, indicates partial degradation of the molecule. In this case, the value for the second signal is shifted towards a higher temperature and is different from the result obtained during the IET measurements. The occurrence of such a difference may be caused by the differences in measurement conditions [22], as in the case of IET tests were performed in an open glass tube, whereas DSC was conducted with samples in aluminium crucibles that were equipped with a single hole in the lid.

### 2.8. Determination of the Energetic Performance—Underwater Explosion Test

**Figure 11:**
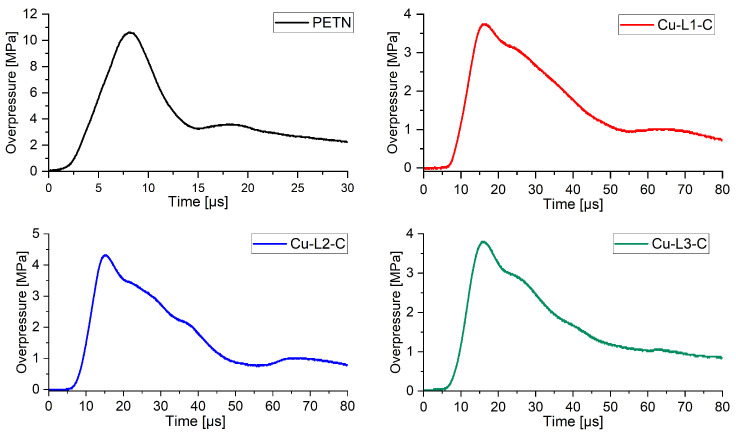
The primary shock wave generated in water by firing detonators filled with 1g of the investigated EMs.

The shock wave in water is generated by the crossing of the detonation wave from the charge into the surrounding medium, where it is further propagated. The secondary pressure wave is associated with the first collapse of the gas bubble. The reference EM (PETN) possesses the highest values of peak overpressure (P_max_) of all the tested compounds (Figure 11).

Significantly lower maximum overpressures were recorded for the perchlorate analogues tested when compared to the equal mass of PETN charge (Figure 11). The highest recorded P_max_ value for 800 mg charges was recorded for Cu-L2-C (40% PETN) and the lowest for Cu-L1-C (35% PETN).

The results of the underwater explosion test are summarised in Table 3. Other data, such as t_b_, E_S_, E_SW_, E_B_ and E_BW_, are collected in Table A9. Based on the measured energies of the shock wave and bubble energies generated in water, total output energy (E) values were calculated for the studied detonators.

The primary shock wave energy (E_SW_), shock energy equivalent (E_s_), bubble gas energy (E_BW_) and bubble gas energy equivalent (E_B_) were calculated from the data recorded by the pressure sensor (Table 3). The highest value of shock wave energy, which is related to the work capacity of the detonation products, was determined for Cu-L2-C (51% PETN). In contrast, the ESWs for Cu-L1-C and Cu-L3-C relative to PETN were 44 and 40%. Conversely, the highest value of bubble gas energy was determined for Cu-L3-C (88% PETN). The total energies, expressed as the sum of primary shock wave energies and the bubble gas energies, were also determined. The total energy values obtained in the study are as follows: Cu-DP-C (69% PETN) > Cu-L3-C (66% PETN) > Cu-L1-C (64% PETN). The differences between the total energy values are not significant, which means that the oxidising anion has a greater influence on the energetic parameters than the ligand present in the coordination compound. This hypothesis is also supported by the fact that only compounds containing perchlorate anion underwent detonation.

In the case of the nitrate analogues, the nature of the primary shock wave generated by the Cu-L1-N, Cu-L2-N and Cu-L3-N series detonators indicates that these tested EMs undergo deflagration. The failure of detonation development for these compounds could be likely related to the higher critical diameter than the diameter of the detonators tested (6.4 mm) or with too weak initiating stimulus. Similarly, the highest values of the first bubble collapse (t_b_) were also achieved for the reference EM. Nevertheless, t_b_ obtained for Cu-L1-C, Cu-L2-C and Cu-L3-C differ from PETN t_b_ by no more than 7%.

## 3. Materials and Methods

All reactants used in the syntheses of ECCs have been used as received without further purification. The purity of the compounds and their suppliers are presented in the appendix (Table A1).

### 3.1. Synthesis

Into a 150 cm^3^ round-bottom flask placed in a water bath, equipped with a mechanical stirrer with a glass stirring rod, the corresponding nitrate or perchlorate salt of the selected transition metal and water were introduced. The salt solution was stirred for 10 min until it was completely dissolved. Then, the addition of ligand in small portions was started, taking place over 45 min, at 15-min intervals (during the addition of further portions of the ligand, a change in the colour of the solution is observed). After the entirety of the ligand was added, the solution was stirred for another 30 min. If a precipitate was present after this time, the solution was filtered gravitationally. To precipitate the product, the solution was transferred to a rotary evaporator and the solvent was evacuated under vacuum until a dry residue remained. The obtained product was washed with ethanol, which was then evacuated at room temperature. Subsequently, the obtained product was dried in an oven at 60 °C for 4 h. For all prepared compounds, the synthesis procedure was the same. The detailed amounts of reagents used are provided in Table A3, Table A4, Table A5 and Table A6. In order to remove unreacted reagents, the compounds obtained were washed three times with acetone (1.5 mL of acetone each) and then left at room temperature until the solvent evaporated completely. The synthesis takes place according to the following reaction:

$$M\left(A\right)x_{1}\;\cdot \;x_{2}\;H_{2}O+n\;L\overset{H_{2}O,\;R.T.}{\to }\left[M{\left(L\right)}_{n}\right]{\left(A\right)}_{x_{1}}$$

where:

M—transition metal, in our case Fe, Ni, Cu or Zn;
L—ligand, in our case EDA, DP, TAEA or TAPA;
x_1_—valency of the transition metal M;
x_2_—hydration number, dependent on the used transition metal salt;
n—number of ligands coordinated to the transition metal atom;

### 3.2. Spectroscopic Investigations

IR spectroscopy was carried out on a Perkin-Elmer Spectrum Two (Waltham, MA, USA) spectrometer, equipped with a universal attenuated total reflectance (UATR) (Single Reflection Diamond module). The spectra of the synthesised coordination compounds were recorded in the range of 650–4000 cm^−1^.

Raman spectra were recorded using an InVia confocal Raman spectrometer from Renishaw (Great Britain) equipped with a DM2500 microscope from Leica (Nussloch, Germany). The excitation source was a helium–neon gas laser (632.8 nm). For measurements, a diffraction grating with 1200 lines/mm was used. During Raman analyses, 1% or 5% of the maximum laser power was used, which was 7.6 mW measured on a 50× objective. To focus on the sample surface, a 50× Leica magnifying lens was used (numerical aperture of the lens N.A. = 0.75), for which the size of the laser beam interacting with the sample is approximately 2 μm. The detector was a camera with a high-resolution CCD (charge-coupled device) matrix. Raman spectra were recorded in the range of 120–3500 cm^−1^. Measurements were carried out with an exposure time of 10 s and an accumulation number of 5. For each sample, measurements were made in at least 3 randomly selected places. The initial analysis of the spectra was carried out using Renishaw’s Wire 3.2 software.

### 3.3. Morphology Investigation—Scanning Electron Microscopy

The morphology of the compounds obtained was determined using scanning electron microscopy (SEM—Inspect S50-FEI and Phenom with EDS detector) allowing measuring the dimensions of morphological features.

### 3.4. X-ray Diffractometry

Powder X-ray diffraction measurements were performed using a Rigaku MiniFlex 600 (Rigaku Co., Tokyo, Japan) diffractometer, equipped with a solid-state silicon strip detector D/teX Ultra. The powder X-ray diffraction (PXRD) patterns were recorded in the doubled scattering angle range of 3–80° (2 θ) using Cu-Kα radiation (λ = 1.5406 Å) operated at 40 mA and 15 kV using Bragg–Brentano geometry with the scanning step size of 0.01°. The exposure time at each point was 1.67 s without sample rotation. For all scans, the IHS slit = 5 mm, Soller slits = 2.5° and DS slit = 1.25° were used.

### 3.5. Elemental Analysis

The elemental analysis investigation was carried out using Elementar’s Vario EL III apparatus. The apparatus works on the principle of catalytic combustion in a tube under oxygenation and high temperature. The combustion gases are purified from interfering gases and then the measured components are separated from each other using adsorbent columns. Determination of the amount of individual components is performed using a thermal conductivity detector (TCD). Combustion is carried out in an atmosphere of oxygen (99.995%), with helium (99.995%) serving as a support and purge gas. Each result is the average obtained from three experimental measurements, for samples of 2.5–3.0 mg each.

### 3.6. Investigation of the Sensitivity to Friction and Impact

The friction and impact sensitivity values of the obtained compounds were determined in accordance with international standards EN 13631-3:2005 [23] using a friction apparatus and EN 13631-4:2002 [24] using a BAM fallhammer, respectively.

### 3.7. Determination of the Ignition/Explosion Temperature (I/ET)

The ignition/explosion temperatures of energetic coordination compounds were determined using an Automatic Explosion Temperature Tester—AET 402 (OZM Research, Bliznovice, Czech Republic). The results obtained represent the average of 5 measurements and the mass of each sample was 50 ± 1 mg. Measurements were carried out in the temperature range of 100–400 °C with a heating rate of 5 °C/min.

### 3.8. Differential Scanning Calorimetry

Differential scanning calorimetry (DSC) analysis of the samples Cu-L2-C and Cu-L3-N was performed using a DSC 3 scanning calorimeter from Mettler-Toledo, operating in the temperature range from 90 °C to 700 °C. Samples for the DSC measurements were sealed in 40 μL standard aluminum crucibles with a single hole punched in the lid. The weight of the analyzed samples was between 1.30 and 1.35 mg for Cu-L2-C and between 1.58 and 1.65 mg for Cu-L3-N. A sample was heated in the temperature range from 20 °C to 350 °C at a heating rate of 5 K/min under N_2_ flow. The measurement was repeated three times for each sample.

### 3.9. Preparation of Test Detonators

**Figure 12:**
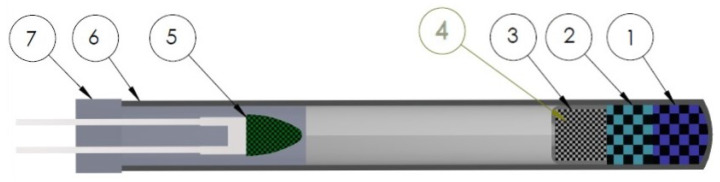
Schematic diagram of detonator where: 1—high-density ECC layer, 2—low-density ECC layer, 3—aluminum cap, 4—lead(II) azide layer, 5—fuse head, 6—Al-Zn alloy shell, 7—rubber shield.

The detonators (Figure 12) were prepared with a one-sided pressing technique. The Al-Zn alloy shells with the following dimensions were used: outer diameter, 6.4 mm; wall thickness, 0.7 mm; length, 62.5 mm. All components were dried for 6 h at 60 °C. The given mass of the EM was poured into a shell and pressed with a hydraulic press via stamp with the force of 1100 N).

Multi-step pressing of detonators provides uniform density across the whole charge. In each case, 800 mg of tested coordination compound was pressed in a two-step process, using 400 mg of the compound during each pressing. In the next step, a 300 mg portion of lead(II) azide was poured inside the detonator and pressed with an aluminium cap, so as to provide the necessary mechanical durability of the detonator. The exact data for the number of pressing operations and the weights for each test batch are given in Table A8. Reference detonators were prepared in a similar manner.

After the pressing process, the 0.2 A fuse heads produced by Nitroerg S.A. were fitted inside, the plastic body was clamped in the detonator, in such a manner as to provide waterproof sealing. As an auxiliary, class 4 PETN was used, according to the MIL-P-387 C standard, produced by SSE Group SA and DS class lead(II) azide according to BN-90 6092-04 standard, produced by Nitroerg S.A.

### 3.10. Determination of the Energetic Performance—Underwater Explosion Test

The underwater explosion tests were performed in accordance with international standard EN 13763-15 [25].

**Figure 13:**
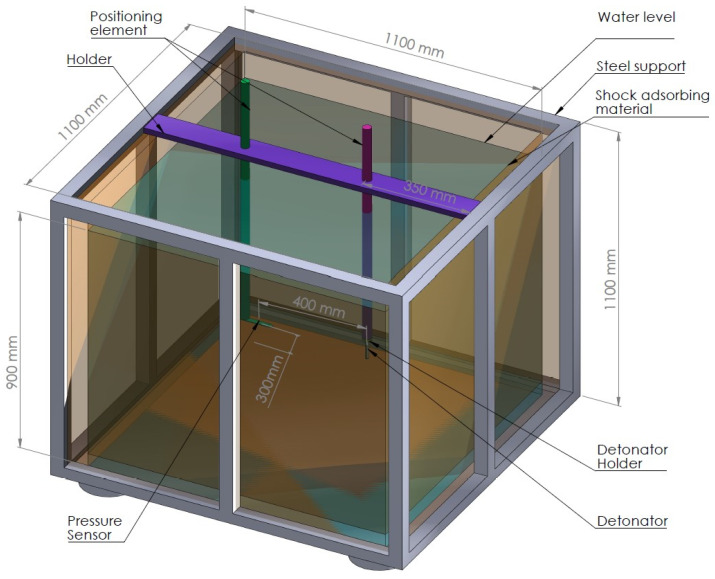
Underwater explosion test set up.

The water tank used for this test (Figure 13) has been built from a stainless steel rack, connected with interior plates of high-density polyurethane, designed to absorb explosion energy and to avoid wave reflection. On the top of the tank, the detonator and pressure sensor positioning rack were placed. Each detonator was placed vertically 550 mm below water level (measured from the centre of the detonator). The tourmaline pressure sensor (PCB Piezotronics 138A10, sensitivity 73.15 mV/MPa, PCB Piezotronics, Depew, NY, USA) was placed 400 mm from the charge axis (measured from the sensitive element of the sensor) and 550 mm below water level. The sensitive element of the sensor was placed at the height of the detonator centre. Sensor data were recorded with an AMC VIBRO CONDITION 8000D (AMC VIBRO, Kraków, Poland) signal conditioner and Textronic TBS2401B digital oscilloscope (Tektronix, Beaverton, OR, USA). The recorded waveforms were analysed with Origin software 2023. All tests were performed 5 times for water temperature in the range of 19.5–20 °C.

Energy equivalence of the shock wave can be defined as:

(1) $$E_{SW}=\frac{4\pi \cdot R_{2}}{\rho_{water}\cdot C_{water}}\int_{t_{0}}^{\theta }p^{2}\;dt$$

where:

*E*_SW_—shock wave energy equivalence (J);
ρ_water_—water density g/cm^3^;
*C*_water_—sound velocity in water;
*R*_2_—distance between detonator and pressure sensor;
θ—(p_max_/e);
*p*—pressure (Pa)

Bubble pulse energy equivalence is related with pulse time by equation:

(2) $$t_{B}=\frac{1.135\cdot \sqrt{\rho_{water}}\cdot \sqrt[{3}]{E_{BW}}}{\sqrt[{6}]{p_{h}^{5}}}=\frac{1.135\cdot \sqrt{\rho_{water}}\cdot \sqrt[{3}]{E_{BW}}}{\sqrt[{6}]{{\left(g\cdot h\cdot \rho_{water}+101,325\right)}^{5}}}$$

where:

*t*_B_—time between pressure peak and first bubble collapse (ms);
*E*_BW_—bubble pulse energy equivalence (J);
*p*_h_—hydrostatic pressure (Pa);
*g*—standard gravity = 9.81 (m/s^2^);
*h*—depth of detonator immersion (m)

Equation (2) for constant conditions may be simplified to:

(3) $$\frac{t_{B}}{K_{1}}=\sqrt[{3}]{E_{BW}}$$

where:

*K*_1_—equal for our system to 23.14·10^−4^, as described with equation:
  (4) $$K_{1}=\frac{1.135\cdot \sqrt{\rho_{water}}}{\sqrt[{6}]{{\left(g\cdot h\cdot \rho_{water}+101,325\right)}^{5}}}$$

The total energy of the explosion is the sum of shock wave energy equivalent and bubble gas energy equivalent:

(5) $$E=E_{SW}+E_{BW}$$

## 4. Conclusions

In conclusion, we have synthesised a series of 32 energetic coordination compounds and shown that their performance in detonators is comparable with PETN (used as the reference EM). Most of these compounds outclass PETN in terms of safety (less sensitive than PETN to impact and to friction), environmental friendliness and stability (no decomposition with release of nitric acid, as in the case of PETN). Due to these compounds being produced mostly from common raw materials of the chemical industry, they are a potential, “green” alternative to nitric acid esters (such as PETN) used in detonators.

Based on their detonation properties, the synthesized ECCs can be viewed largely as secondary energetic materials. This is well-evidenced by the ECCs requiring an initial charge of primary EM to achieve detonation and in some cases undergoing deflagration if the primary EM charge was not sufficiently potent. Interestingly, despite the compounds tested in mock detonators being used in their unpurified forms (containing significant amounts of relatively inert by-products), achieved bubble energies were comparable to those achieved by PETN-based mock detonators, making the ECCs highly promising materials.

In terms of future work on this class of compounds, development of cost-efficient and reliable purification methods for each of the ECCs, followed by a more in-depth study of their energetic properties, appears to be the most important aspect. In terms of utilising the produced ECCs in initiating systems, their deflagration to detonation parameters should be investigated, along with fine-tuning both the morphology (e.g., via recrystallization from common solvents) and density of the ECCs, compared to what we have used in the initial study in mock detonators.

## Figures and Tables

**Table 1:** Elemental analysis results for purified Cu-L2-C.

Compound	Composition [%]		Gross Formula
	**Calculated**	**Found**	
	C—10.90	C—10.70	
${\left[Cu{\left(ClO_{4}\right)}_{2}\right]}_{4}{\left[C_{3}N_{2}H_{1}0\right]}_{1.3}{\left[NH_{3}\right]}_{0.1}{\left[H_{2}O\right]}_{0.4}$	H—4.13	H—4.16	$Cu_{4}Cl_{8}C_{3.9}H_{14.1}N_{2.7}O_{8.4}$
	N—9.89	N—10.16	

**Table 2:** Ignition/explosion temperature for obtained ECCs. The results obtained represent the average of 5 measurements.

Oxidising Anion: NO_3_^−^		Oxidising Anion: ClO_4_^−^	
**Sample Code**	**I/ET [°C]**	**Sample Code**	**I/ET [°C]**
Fe-L1-N	181 ± 1	Fe-L1-C	165 ± 1
Ni-L1-N	260 ± 3	Ni-L1-C	224 ± 3
Cu-L1-N	224 ± 2	Cu-L1-C	259 ± 2
Zn-L1-N	292 ± 2	Zn-L1-C	183 ± 1
Fe-L2-N	214 ± 3	Fe-L2-C	205 ± 3
Ni-L2-N	256 ± 1	Ni-L2-C	261 ± 1
Cu-L2-N	210 ± 2	Cu-L2-C	240 ± 2
Zn-L2-N	300 ± 1	Zn-L2-C	364 ± 2
Fe-L3-N	178 ± 1	Fe-L3-C	158 ± 2
Ni-L3-N	248 ± 2	Ni-L3-C	280 ± 1
Cu-L3-N	211 ± 2	Cu-L3-C	228 ± 2
Zn-L3-N	280 ± 2	Zn-L3-C	158 ± 1
Fe-L4-N	208 ± 1	Fe-L4-C	199 ± 1
Ni-L4-N	231 ± 1	Ni-L4-C	244 ± 1
Cu-L4-N	224 ± 1	Cu-L4-C	233 ± 2
Zn-L4-N	253 ± 2	Zn-L4-C	362 ± 2

**Table 3:** Values of the shock wave parameters: P_max_, E for the reference EM and obtained ECCs.

Sample Code	m [g]	P_max_ [MPa]	E [J]
PETN	0.6	9.67 (±0.34)	1399 (±16)
	0.8	10.58 (±0.19)	1696 (±35)
	1.0	11.68 (±0.25)	1963 (±18)
Cu-L1-C	0.8	3.71 (±0.02)	1078 (±17)
Cu-L2-C	0.8	4.20 (±0.08)	1167 (±20)
Cu-L3-C	0.8	3.75 (±0.04)	1117 (±8)

**Figure A1:**
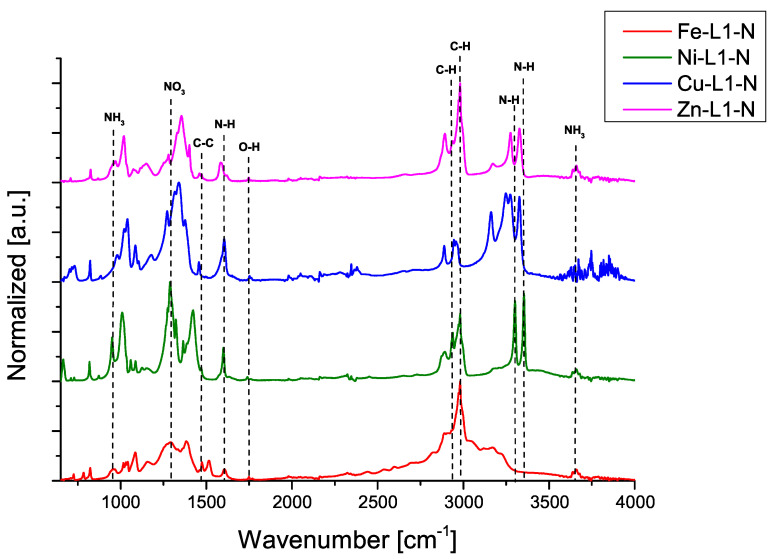
IR-ATR spectra for energetic coordination compounds containing 1,2-ethylenediamine and nitrate salts.

**Figure A2:**
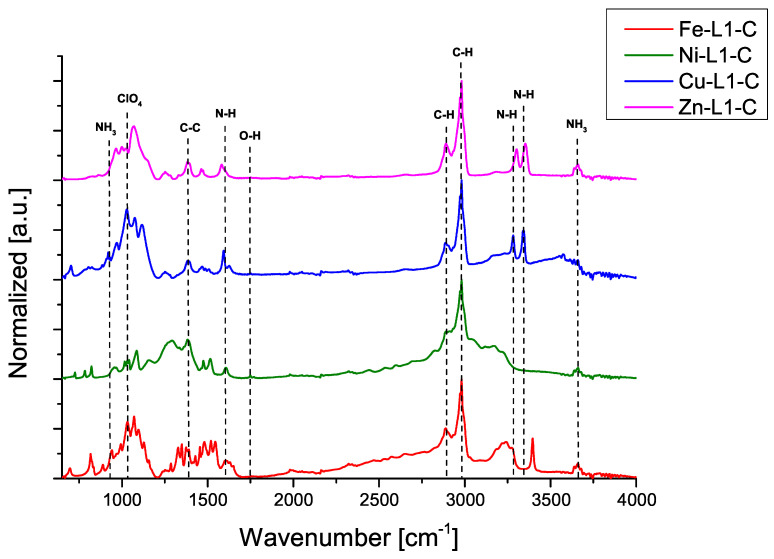
IR-ATR spectra for energetic coordination compounds containing 1,2-ethylenediamine and perchlorate salts.

**Figure A3:**
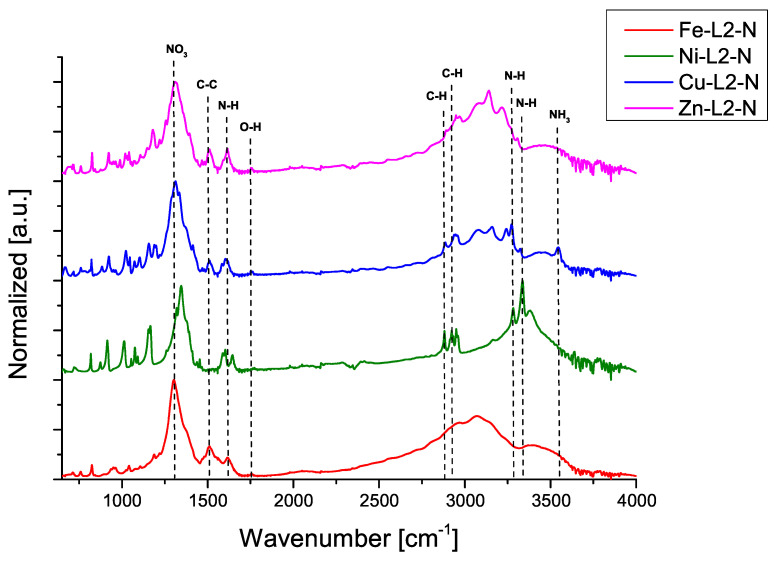
IR-ATR spectra for energetic coordination compounds containing 1,3-diaminopropane and nitrate salts.

**Figure A4:**
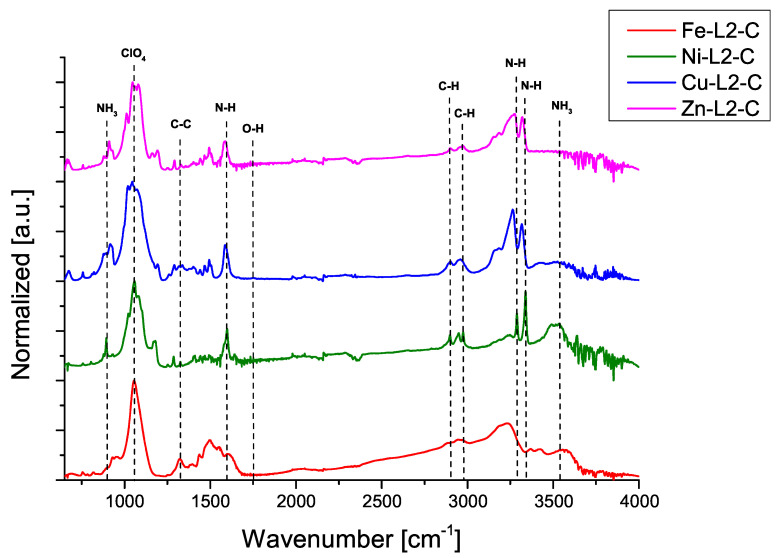
IR-ATR spectra for energetic coordination compounds containing 1,3-diaminopropane and perchlorate salts.

**Figure A5:**
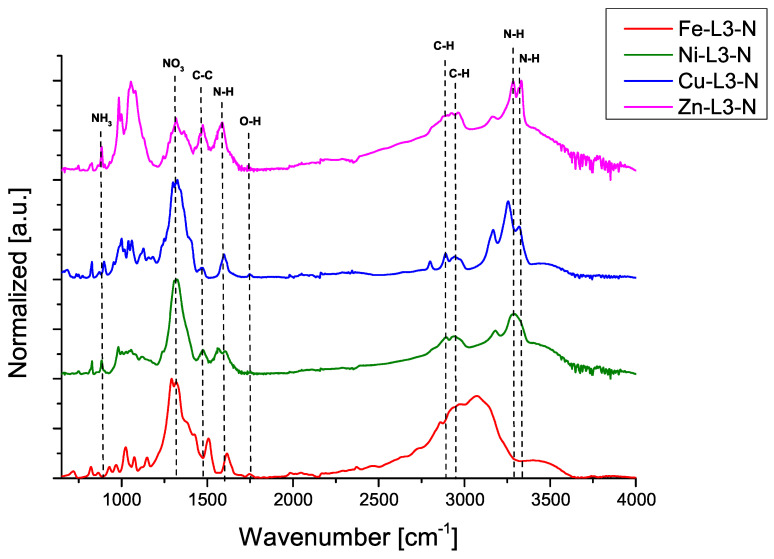
IR-ATR spectra for energetic coordination compounds containing tris(2-aminoethyl)amine and nitrate salts.

**Figure A6:**
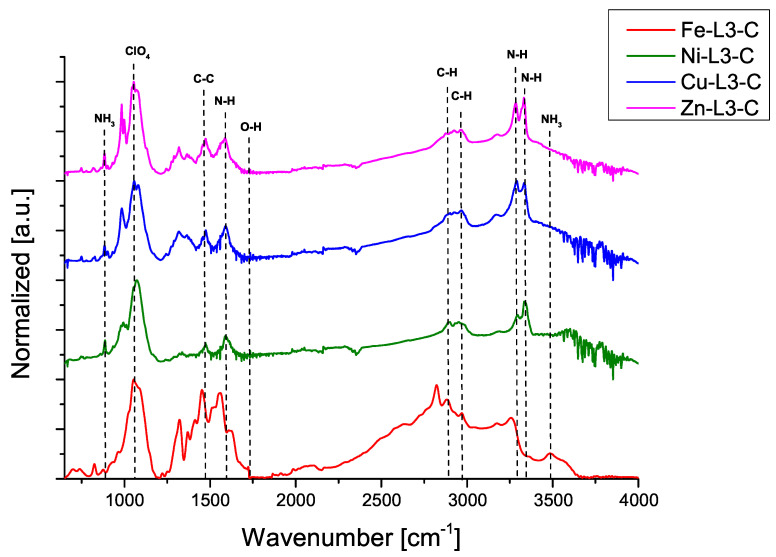
IR-ATR spectra for energetic coordination compounds containing tris(2-aminoethyl)amine and perchlorate salts.

**Figure A7:**
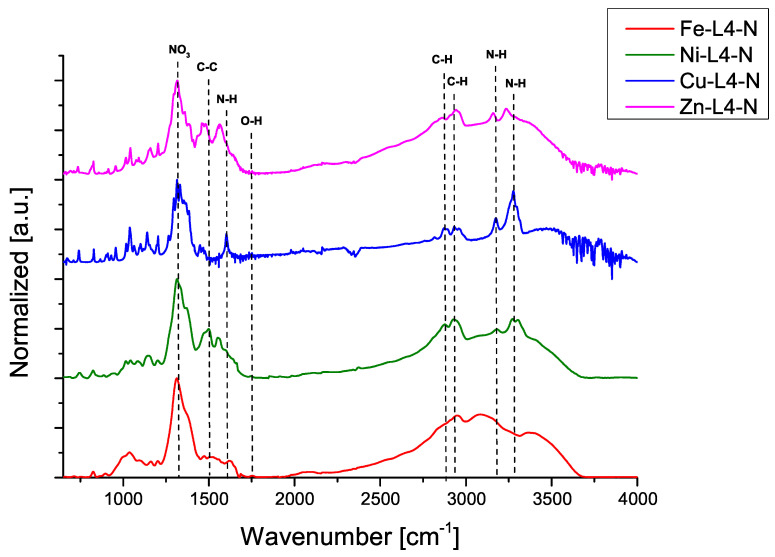
IR-ATR spectra for energetic coordination compounds containing tris(3-aminopropyl)amine and nitrate salts.

**Figure A8:**
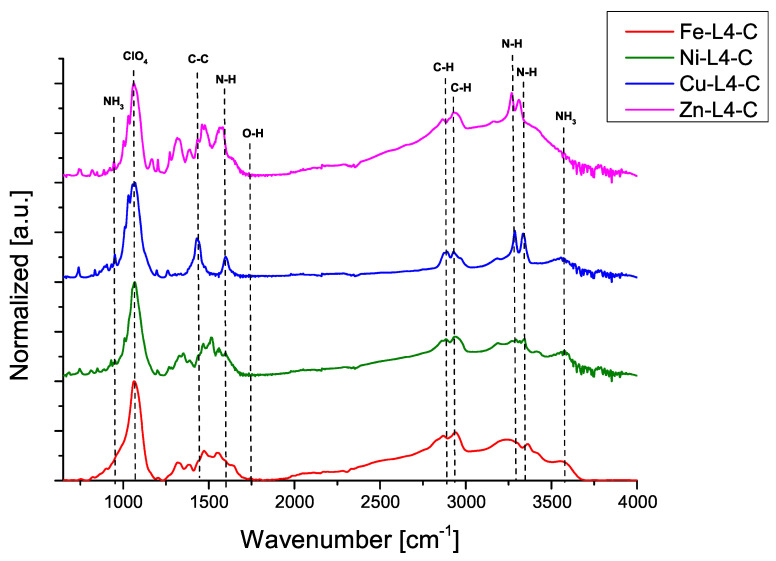
IR-ATR spectra for energetic coordination compounds containing tris(3-aminopropyl)amine and perchlorate salts.

## Data Availability

Data is available on reasonable request from the authors.

## Abbreviations

The following abbreviations are used in this manuscript:

DSC	Differential scanning calorimetry
ECCs	Energetic coordination compounds
EMs	Energetic materials
I/ET	Ignition/explosion temperature
IR-ATR	Attenuated Total Reflectance spectroscopy
PETN	Pentaerythritol tetranitrate
SEM	Scanning electron microscope
XRD	X-ray diffraction

## Appendix A

**Table A1:** Chemicals used in the syntheses of ECCs.

Chemical	Purity [%]	Source
Iron(III) nitrate nonahydrate	≥98	Sigma-Aldrich
Nickel(II) nitrate hexahydrate	≥98.5	Sigma-Aldrich
Copper(II) nitrate hemi(pentahydrate)	>98	Sigma-Aldrich
Zinc nitrate hexahydrate	>98	Sigma-Aldrich
Iron(II) perchlorate hydrate	>98	Sigma-Aldrich
Nickel(II) perchlorate hexahydrate	>98	Sigma-Aldrich
Copper(II) perchlorate hexahydrate	>98	Sigma-Aldrich
Zinc perchlorate hexahydrate	>98	Sigma-Aldrich
1,2-ethylenediamine	>99	Chemsolve
1,3-diaminopropane	>98	TCI
Tris(2-aminoethyl)amine	>98	TCI
Tris(3-aminopropyl)amine	>97	TCI
Ethanol 96%	cz. d. a.	Stanlab

**Table A2:** Sample codes corresponding to synthesized energetic coordination compounds in configuration M-L-A (where M—metal; L—ligand; A—oxidizing anion; N indicates NO_3_^−^, C indicates ClO_4_^−^).

Metal	Ligand			
	**EDA**	**DP**	**TAEA**	**TAPA**
Fe	Fe-L1-N	Fe-L2-N	Fe-L3-N	Fe-L4-N
	Fe-L1-C	Fe-L2-C	Fe-L3-C	Fe-L4-C
Ni	Ni-L1-N	Ni-L2-N	Ni-L3-N	Ni-L4-N
	Ni-L1-C	Ni-L2-C	Ni-L3-C	Ni-L4-C
Cu	Cu-L1-N	Cu-L2-N	Cu-L3-N	Cu-L4-N
	Cu-L1-C	Cu-L2-C	Cu-L3-C	Cu-L4-C
Zn	Zn-L1-N	Zn-L2-N	Zn-L3-N	Zn-L4-N
	Zn-L1-C	Zn-L2-C	Zn-L3-C	Zn-L4-C

**Table A3:** Amount of reagents used for synthesis of ECCs based on 1,2-ethylenediamine and yields achieved.

Metal Salt	Amount of Reagent			Yield [%]
	**Metal Salt**	**Ligand**	**H_2_O**	
$Fe{\left(NO_{3}\right)}_{3}\cdot 9H_{2}O$	0.96 g (2.39 mmol)	0.48 g (7.11 mmol)	23.7 cm^3^	75
$Ni{\left(NO_{3}\right)}_{2}\cdot 6H_{2}O$	0.80 g (2.76 mmol)	0.55 g (8.27 mmol)	27.6 cm^3^	77
$Cu{\left(NO_{3}\right)}_{2}\cdot 2.5H_{2}O$	0.76 g (3.25 mmol)	0.43 g (9.75 mmol)	32.5 cm^3^	76
$Zn{\left(NO_{3}\right)}_{2}\cdot 6H_{2}O$	0.81 g (2.71 mmol)	0.54 g (8.12 mmol)	27.1 cm^3^	61
$Fe{\left(ClO_{4}\right)}_{3}\cdot xH_{2}O$	0.70 g (1.87 mmol)	0.38 g (5.62 mmol)	18.7 cm^3^	85
$Ni{\left(ClO_{4}\right)}_{2}\cdot 6H_{2}O$	0.84 g (2.29 mmol)	0.46 g (6.86 mmol)	26.1 cm^3^	91
$Cu{\left(ClO_{4}\right)}_{2}\cdot 6H_{2}O$	0.97 g (2.61 mmol)	0.35 g (7.84 mmol)	26.1 cm^3^	90
$Zn{\left(ClO_{4}\right)}_{2}\cdot 6H_{2}O$	0.84 g (2.25 mmol)	0.45 g (6.75 mmol)	22.5 cm^3^	93

**Table A4:** Amount of reagents used for synthesis of ECCs based on 1,3-diaminopropane and yields achieved.

Metal Salt	Amount of Reagent			Yield [%]
	**Metal Salt**	**Ligand**	**H_2_O**	
$Fe{\left(NO_{3}\right)}_{3}\cdot 9H_{2}O$	0.64 g (1.60 mmol)	0.28 g (3.19 mmol)	16.0 cm^3^	79
$Ni{\left(NO_{3}\right)}_{2}\cdot 6H_{2}O$	0.57 g (1.95 mmol)	0.34 g (3.90 mmol)	19.5 cm^3^	82
$Cu{\left(NO_{3}\right)}_{2}\cdot 2.5H_{2}O$	0.61 g (2.63 mmol)	0.30 g (5.25 mmol)	26.3 cm^3^	81
$Zn{\left(NO_{3}\right)}_{2}\cdot 6H_{2}O$	0.57 g (1.92 mmol)	0.33 g (3.85 mmol)	19.2 cm^3^	79
$Fe{\left(ClO_{4}\right)}_{3}\cdot xH_{2}O$	0.63 g (1.68 mmol)	0.29 g (3.36 mmol)	16.8 cm^3^	97
$Ni{\left(ClO_{4}\right)}_{2}\cdot 6H_{2}O$	0.62 g (1.70 mmol)	0.30 g (3.40 mmol)	17.0 cm^3^	84
$Cu{\left(ClO_{4}\right)}_{2}\cdot 6H_{2}O$	0.71 g (1.93 mmol)	0.22 g (3.85 mmol)	19.3 cm^3^	85
$Zn{\left(ClO_{4}\right)}_{2}\cdot 6H_{2}O$	0.63 g (1.68 mmol)	0.29 g (3.36 mmol)	16.8 cm^3^	82

**Table A5:** Amount of reagents used for synthesis of ECCs based on tris(2-aminoethyl)amine and yields achieved.

Metal Salt	Amount of Reagent			Yield [%]
	**Metal Salt**	**Ligand**	**H_2_O**	
$Fe{\left(NO_{3}\right)}_{3}\cdot 9H_{2}O$	0.76 g (1.87 mmol)	0.58 g (3.74 mmol)	18.7 cm^3^	87
$Ni{\left(NO_{3}\right)}_{2}\cdot 6H_{2}O$	0.61 g (2.10 mmol)	0.66 g (4.21 mmol)	21.0 cm^3^	89
$Cu{\left(NO_{3}\right)}_{2}\cdot 2.5H_{2}O$	0.61 g (2.61 mmol)	0.54 g (5.23 mmol)	26.1 cm^3^	92
$Zn{\left(NO_{3}\right)}_{2}\cdot 6H_{2}O$	0.62 g (2.08 mmol)	0.65 g (4.15 mmol)	20.8 cm^3^	86
$Fe{\left(ClO_{4}\right)}_{3}\cdot xH_{2}O$	0.58 g (1.55 mmol)	0.48 g (3.09 mmol)	15.5 cm^3^	91
$Ni{\left(ClO_{4}\right)}_{2}\cdot 6H_{2}O$	0.66 g (1.82 mmol)	0.57 g (3.64 mmol)	18.2 cm^3^	90
$Cu{\left(ClO_{4}\right)}_{2}\cdot 6H_{2}O$	0.81 g (2.19 mmol)	0.45 g (4.37 mmol)	21.9 cm^3^	94
$Zn{\left(ClO_{4}\right)}_{2}\cdot 6H_{2}O$	0.67 g (1.80 mmol)	0.56 g (3.59 mmol)	18.0 cm^3^	88

**Table A6:** Amount of reagents used for synthesis of ECCs based on tris(3-aminopropyl)amine and yields achieved.

Metal Salt	Amount of Reagent			Yield [%]
	**Metal Salt**	**Ligand**	**H_2_O**	
$Fe{\left(NO_{3}\right)}_{3}\cdot 9H_{2}O$	0.52 g (1.28 mmol)	0.52 g (2.56 mmol)	12.8 cm^3^	95
$Ni{\left(NO_{3}\right)}_{2}\cdot 6H_{2}O$	0.44 g (1.50 mmol)	0.61 g (3.00 mmol)	15.0 cm^3^	86
$Cu{\left(NO_{3}\right)}_{2}\cdot 2.5H_{2}O$	0.48 g (2.07 mmol)	0.56 g (4.13 mmol)	20.7 cm^3^	98
$Zn{\left(NO_{3}\right)}_{2}\cdot 6H_{2}O$	0.44 g (1.48 mmol)	0.60 g (2.97 mmol)	14.8 cm^3^	89
$Fe{\left(ClO_{4}\right)}_{3}\cdot xH_{2}O$	0.50 g (1.34 mmol)	0.54 g (2.67 mmol)	13.4 cm^3^	91
$Ni{\left(ClO_{4}\right)}_{2}\cdot 6H_{2}O$	0.49 g (1.35 mmol)	0.55 g (2.69 mmol)	13.5 cm^3^	88
$Cu{\left(ClO_{4}\right)}_{2}\cdot 6H_{2}O$	0.60 g (1.61 mmol)	0.44 g (3.22 mmol)	16.1 cm^3^	77
$Zn{\left(ClO_{4}\right)}_{2}\cdot 6H_{2}O$	0.50 g (1.34 mmol)	0.54 g (2.67 mmol)	13.4 cm^3^	91

**Table A7:** Friction and impact sensitivity values for synthesized ECCs.

Oxidizing Anion: NO_3_^−^			Oxidizing Anion: ClO_4_^−^		
**Sample Code**	**FS [N]**	**IS [J]**	**Sample Code**	**FS [N]**	**IS [J]**
Fe-L1-N	84	>50	Fe-L1-C	108	>50
Ni-L1-N	128	10	Ni-L1-C	168	3
Cu-L1-N	112	35	Cu-L1-C	96	<1
Zn-L1-N	80	40	Zn-L1-C	144	2
Fe-L2-N	108	10	Fe-L2-C	128	2
Ni-L2-N	64	20	Ni-L2-C	112	5
Cu-L2-N	80	35	Cu-L2-C	60	7.5
Zn-L2-N	96	25	Zn-L2-C	216	5
Fe-L3-N	96	>50	Fe-L3-C	80	3
Ni-L3-N	168	>50	Ni-L3-C	168	5
Cu-L3-N	108	>50	Cu-L3-C	72	<1
Zn-L3-N	192	40	Zn-L3-C	160	5
Fe-L4-N	60	>50	Fe-L4-C	84	5
Ni-L4-N	89	>50	Ni-L4-C	128	7.5
Cu-L4-N	144	>50	Cu-L4-C	288	1
Zn-L4-N	84	>50	Zn-L4-C	216	>50

**Table A8:** Number of pressing operations for preparation of detonators.

Type	Secondary EM	Mass mg	Steps	Primary EM	Mass mg	Steps
Z1	Cu-L1-N	800	2	PbN_6_	300	1
Z2	Cu-L1-C	800	2	PbN_6_	300	1
Z3	Cu-L2-N	800	2	PbN_6_	300	1
Z4	Cu-L2-C	800	2	PbN_6_	300	1
Z5	Cu-L3-N	800	2	PbN_6_	300	1
Z6	Cu-L3-C	800	2	PbN_6_	300	1

**Table A9:** Values of the: t_b_, E_S_, E_SW_, E_B_, E_BW_ for reference EMs and containing ECCs.

Sample Code	m [g]	t _b_ [ms]	E_S_·10^8^ [Pa^2^s]	E_SW_ [J]	E_B_·10^−6^ [s^3^]	E_BW_ [J]
PETN	0.6	21.21 (±0.11)	4.63 (±0.11)	629 (±15)	9.54 (±0.13)	770 (±10)
	0.8	22.44 (±0.10)	5.77 (±0.20)	785 (±27)	11.30 (±0.16)	911 (±13)
	1.0	23.43 (±0.21)	6.80 (±0.21)	925 (±29)	12.86 (±0.34)	1038 (±28)
Cu-L1-C	0.8	20.86 (±0.04)	2.54 (±0.04)	345 (±5)	9.08 (±0.19)	733 (±15)
Cu-L2-C	0.8	21.16 (±0.15)	2.95 (±0.06)	402 (±9)	9.48 (±0.20)	765 (±16)
Cu-L3-C	0.8	21.51 (±0.04)	2.31 (±0.05)	314 (±7)	9.95 (±0.06)	803 (±5)
